# Hybrid machine learning approach for predicting compressive strength of sustainable concrete incorporating palm oil fuel ash

**DOI:** 10.1038/s41598-026-46190-w

**Published:** 2026-05-01

**Authors:** Ramin Kazemi, Mohammad Azimi Pour, Amir H. Gandomi

**Affiliations:** 1 Department of Civil Engineering, Hakim Sabzevari University, Sabzevar, Iran; 2 Department of Civil, Architectural and Environmental Engineering, Missouri University of Science and Technology, Rolla, MO 65401 USA; 3 Faculty of Engineering & Information Technology, University of Technology Sydney, Ultimo, 2007 Australia; 4 University Research and Innovation Center (EKIK), Obuda University, Budapest, 1034 Hungary

**Keywords:** Prediction, Compressive strength, Palm oil fuel ash, Sustainable concrete, Artificial intelligence techniques, Engineering, Materials science

## Abstract

Accurate prediction of compressive strength is important for the efficient design of sustainable concrete incorporating palm oil fuel ash (POFA). This study developed a hybrid artificial neural network model optimized using biogeography based optimization (ANN-BBO) to predict the compressive strength of POFA-based concrete using a compiled literature database of 469 mixtures collected from 22 independent studies. The proposed framework was intended to improve predictive performance relative to a conventional standalone ANN by combining nonlinear learning capability with metaheuristic parameter optimization. The models were developed using six mixture design variables: cement content, POFA content, superplasticizer dosage, coarse-to-fine aggregate ratio, water-to-binder ratio, and curing age. Model performance was evaluated using multiple statistical measures, including the coefficient of determination (R^2^) and root mean square error (RMSE). For model development, the dataset was divided into training, validation, and testing sets using a 70/15/15 ratio. In addition, model performance was further examined using tenfold cross-validation and bootstrap resampling. Among the evaluated models, ANN-BBO showed the best overall predictive performance, achieving an R^2^ of 0.983 and a mean absolute error of 2.28 MPa, while also performing favorably compared with the standalone ANN and related models reported in previous studies. The cross-validation results showed that ANN-BBO achieved a mean R^2^ of 0.954 and a mean RMSE of 4.658 MPa, compared with 0.907 and 6.728 MPa for the ANN model. In addition, more than 60% of the ANN-BBO prediction errors fell within the range of [− 5%, 5%], whereas the corresponding proportion for the ANN model was about 39%, indicating more consistent predictive performance of the hybrid model. Interpretability analysis further indicated that curing age and water-to-binder ratio were the most influential variables governing compressive strength prediction. Overall, the results suggest that the ANN-BBO model may provide a useful tool for rapid strength estimation and preliminary evaluation of concrete containing POFA mixtures within the scope of the compiled dataset.

**Supplementary Information:**

The online version contains supplementary material available at 10.1038/s41598-026-46190-w.

## Introduction

### Background and literature review

**Fig. 1:**
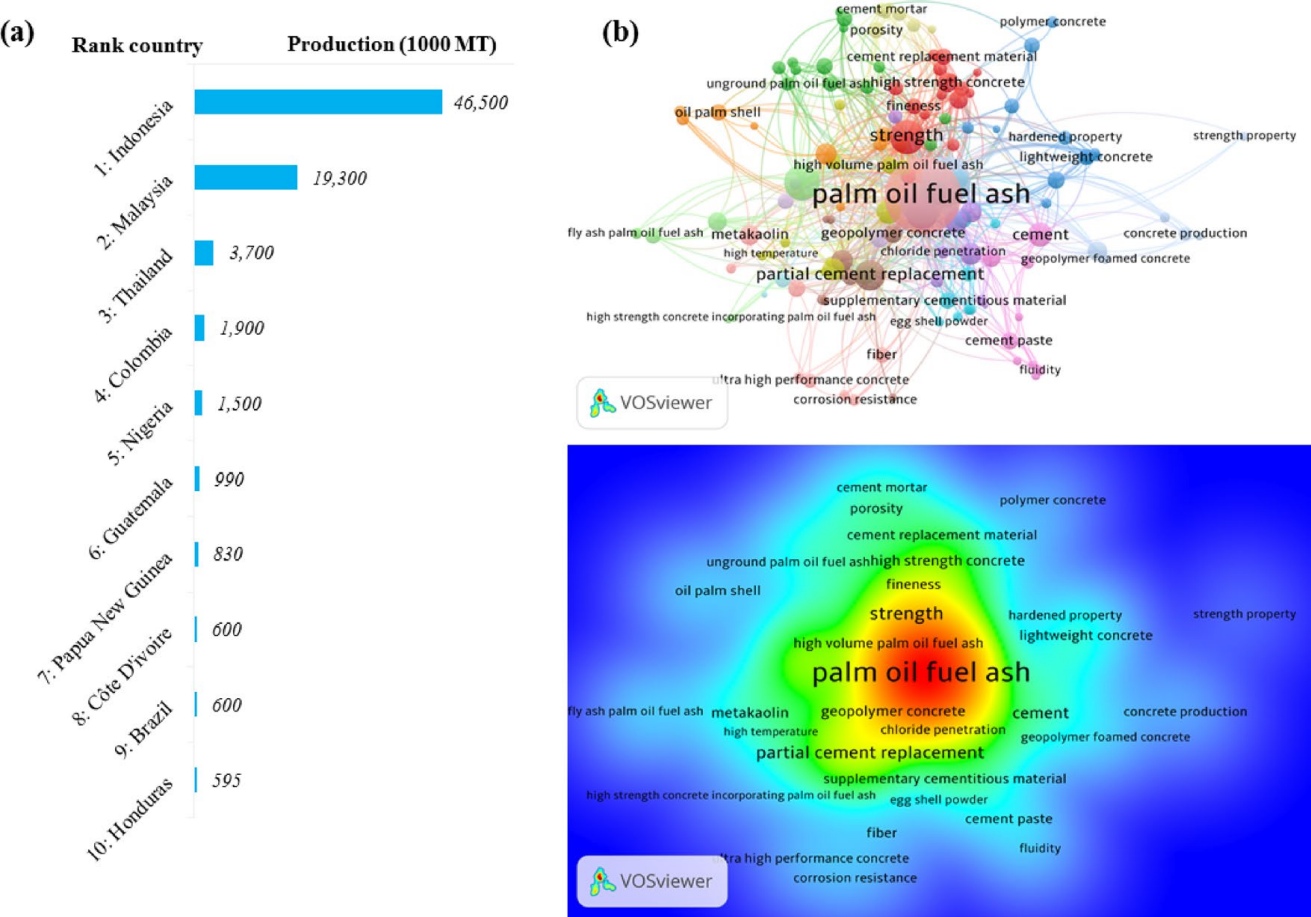
(**a**) Global palm oil production distribution by country in 2024, prepared by the authors using Microsoft Excel 2016 based on data reported in Ref.^21^. (**b**) Bibliometric network and density visualization of palm oil fuel ash research themes, generated by the authors using VOSviewer version 1.6.18^27^.

Concrete is one of the most widely used construction materials worldwide and is essential to modern construction. Nevertheless, the increasing demand for concrete has been accompanied by a rapid rise in cement consumption, which imposes a significant environmental burden because cement production is highly energy-intensive and contributes approximately 5–8% of global anthropogenic CO_2_ emissions^1–3^. As a result, supplementary cementitious materials (SCMs) have received increasing attention as partial cement replacements that can reduce clinker consumption while maintaining or enhancing concrete performance^4–9^. Materials such as fly ash, slag, rice husk ash, silica fume, and natural pozzolans have shown considerable potential in this regard^5,10–16^. Among them, palm oil fuel ash (POFA) has emerged as a promising SCM owing to its abundant availability in palm-oil-producing regions and its potential role in both waste utilization and sustainable concrete production^17–23^. As shown in Fig. 1, the geographical concentration of palm-oil production provides a substantial and continuous source of POFA, while the bibliometric mapping highlights growing research attention toward its application in concrete, especially in relation to partial cement replacement, strength, and durability. Following suitable processing to improve its fineness and reactivity, POFA can be incorporated effectively into cementitious materials while retaining its environmental advantages^24–26^.

Previous studies have shown that POFA can affect concrete performance through its influence on hydration, pore structure, durability, and strength, mainly because of its pozzolanic reactivity and the changes in fineness and surface area achieved after processing^28,29^. Among these properties, compressive strength is the most important performance indicator because it is widely used in mixture design, structural assessment, and quality control of concrete^26,30–32^. In a study, Tangchirapat et al.^33^ evaluated 10–30% cement replacement in high-strength concrete and measured compressive strength, drying shrinkage, water permeability, and sulfate resistance. Their results showed that the 20% mixture achieved about 70 MPa at 90 days and displayed lower shrinkage and permeability, consistent with the formation of secondary C–S–H. In POFA-based systems, compressive strength is governed by several interacting factors, including replacement level, particle fineness, water-to-binder ratio, aggregate proportion, admixture dosage, and curing age. As a result, the influence of POFA on strength becomes highly nonlinear and difficult to capture using conventional empirical approaches alone, which creates a strong motivation for applying artificial intelligence (AI)-based prediction methods.

In recent years, AI techniques have been increasingly adopted to predict the mechanical properties of sustainable concrete, offering substantial reductions in time, cost, and experimental effort. Models such as artificial neural networks (ANN), support vector machines (SVM), gradient boosting algorithms, and gene expression programming have shown strong predictive capability for concretes incorporating SCMs^34–41^. Given the growing interest in POFA as a sustainable SCM and its influence on concrete performance, accurate prediction of the compressive strength of POFA-modified concrete has become increasingly important^19^. Safiuddin et al.^42^ developed an ANN model to predict the 28-day compressive strength of self-consolidating high-strength concrete containing POFA using 20 mixtures with POFA contents ranging from 0 to 30% and water-to-binder ratios of 0.25–0.40. Their results showed that the ANN model with two hidden layers had good capability for predicting 28-day compressive strength. A more comprehensive study was later reported by Ali et al.^43^, who compiled a dataset of 407 samples and evaluated several machine learning techniques, including ANN, bagging, least-squares SVM, GEP, XGB, LGBM, and a hybrid XGB-LGBM model, for predicting the compressive strength of POFA-modified concrete. Their results showed that the hybrid XGB-LGBM model achieved the highest R^2^ of 0.976 and the lowest RMSE, followed by the ANN model with an R^2^ of 0.968. Despite these promising results, many existing studies are still limited by relatively small datasets, narrow mixture ranges, or reliance on standalone models without optimization.

## Research gaps and objectives

A review of the existing literature indicates that several gaps remain in AI-based prediction of the compressive strength of concrete containing POFA. First, although hybrid AI models have shown promising performance in many engineering applications, most of the studies reported for POFA concrete strength prediction have relied primarily on standalone models. Hybrid models may offer advantages by improving the search process for optimal model parameters and reducing the risk of local minima or unstable training outcomes. Second, previous studies have generally provided limited attention to more rigorous model evaluation frameworks, such as three-way data splitting for training, validation, and testing, together with cross-validation procedures that can provide a broader view of model stability. Third, comparative assessment against previously published models remains important for placing newly developed models in context and for evaluating whether they offer meaningful improvement under similar input conditions.

To address these gaps, this study compiles a comprehensive database of 469 concrete mixtures extracted from 22 published studies, covering a wide range of POFA contents, mixture proportions, and curing ages. Using this dataset, two predictive approaches were developed: ANN and a hybrid ANN enhanced with biogeography-based optimization (ANN-BBO). To evaluate their performance, multiple statistical indicators, sensitivity analyses, and reliability assessments were employed. The models were evaluated using multiple statistical indicators, including R^2^, MAE, RMSE, RRMSE, VAF, and a10-index, together with three-way data splitting, k-fold cross-validation, bootstrap resampling, and interpretability analyses. In addition, the performance of the proposed models was compared with that of previously reported AI-based models developed for compressive strength prediction of POFA concretes. Overall, this study aims to provide a more systematic framework for compressive strength prediction and to support the efficient evaluation of sustainable concrete mixtures incorporating POFA.

## Data collection and analysis

**Fig. 2:**
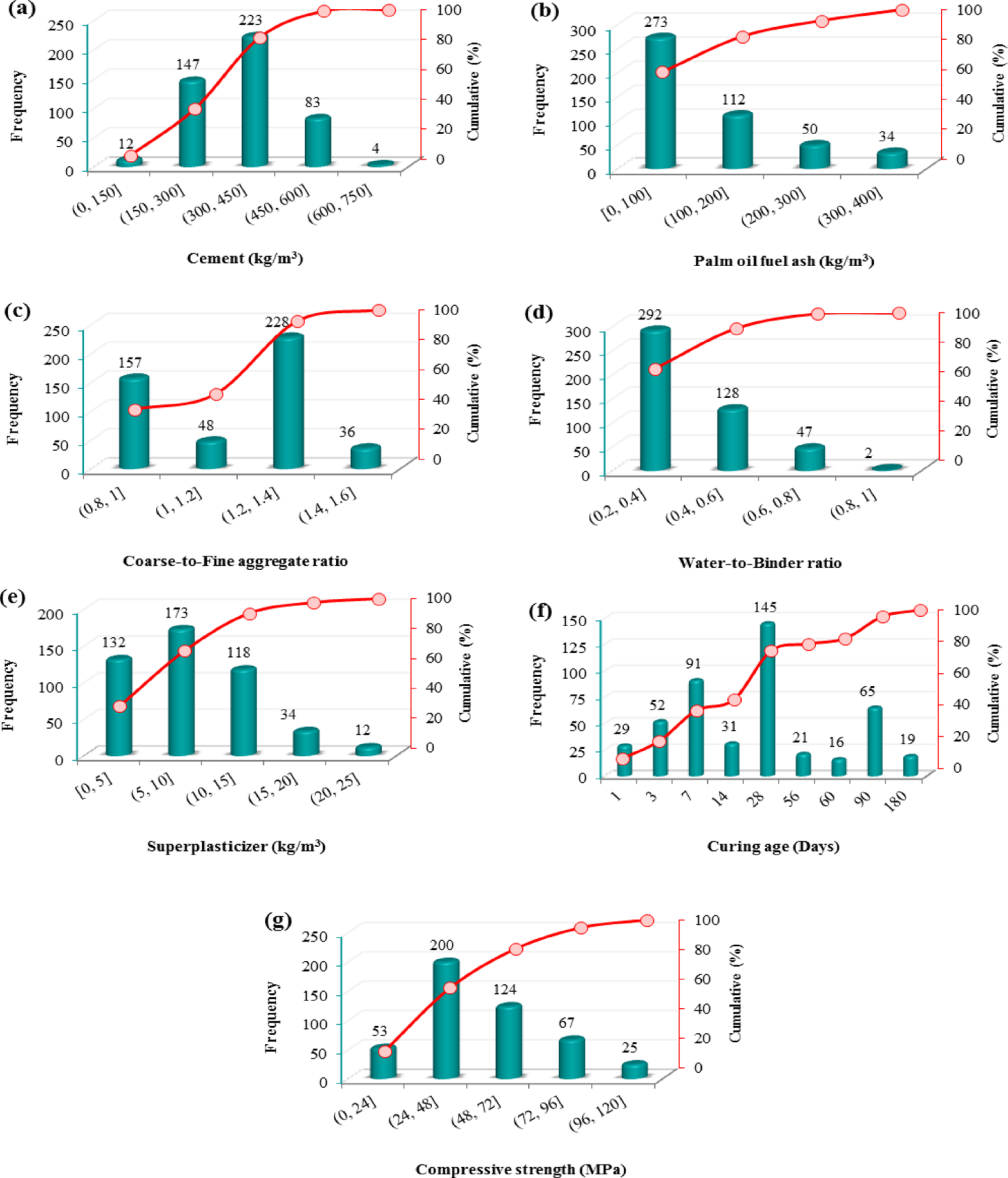
Histograms of the frequency of input (**a**–**f**) and output (**g**) variables.

This study compiled 469 concrete mixtures from 22 publications^19,20,22,23,25,42,44–59^ to develop AI models for predicting the compressive strength of concrete containing POFA. To ensure database quality, a screening and preprocessing procedure was applied during data collection. Only studies with sufficient input and output information for compressive strength modeling were included. Samples with incomplete records, insufficient experimental details, or duplicate entries were excluded. Table 1 reports each paper’s contribution to the dataset. The inputs were cement content (C), POFA content, superplasticizer dosage (SP), the coarse-to-fine aggregate ratio (CA/FA), the water-to-binder ratio (W/B), and curing age (Age). The output was compressive strength (CS). The input variables were selected based on their physical relevance to compressive strength, their consistent availability across the collected literature sources, and their use in recent related prediction studies. Only parameters that were commonly reported in the source publications were retained to ensure adequate database size and completeness. This selection strategy allowed the development of a practically usable model while preserving comparability with previously published work. Figure 2 illustrates histograms of the inputs and CS in different ranges: over 80% of the mixtures have C, POFA, and SP within (0, 450], [0, 200], and [0, 15] kg/m^3^, respectively; most W/B values fall between 0.20 and 0.60; testing ages are mostly at 3, 7, 28, and 90 days; and about 70% of CS values lie in (24, 72] MPa. Table 2 summarizes descriptive statistics (minimum, maximum, mean, standard deviation, kurtosis, and skewness) for all variables. The database used in this study is provided in the supplementary file. However, because the database was compiled from multiple independent studies, CS measurements may not be fully harmonized across sources with respect to specimen shape/size, testing standards, curing regimes, and POFA pre-treatment, especially when such metadata were unavailable or inconsistently reported; therefore, inter-study variability attributable to these factors should be considered a limitation of the dataset and the resulting models.

**Table 1:** Details of the extracted dataset.

No. ID	Authors [Ref.]	Year	No. data	Data contribution rate (%)
1	Sata, et al.^51^	2004	16	3.41
2	Jaturapitakkul, et al.^23^	2007	13	2.77
3	Chindaprasirt, et al.^22^	2007	8	1.7
4	Ahmad, et al.^54^	2008	20	4.26
5	Sata and Jaturapitakkul^45^	2010	72	15.35
6	Tangchirapat and Jaturapitakkul^46^	2010	28	5.97
7	Johari, et al.^55^	2012	16	3.41
8	Awal and Shehu^50^	2013	4	0.85
9	Alsubari, et al.^57^	2014	5	1.07
10	Awal and Shehu^59^	2014	8	1.7
11	Alsubari, et al.^56^	2015	30	6.4
12	Mohammadhosseini, et al.^58^	2015	36	7.68
13	Zeyad, et al.^44^	2016	36	7.68
14	Ranjbar, et al.^25^	2016	24	5.12
15	Safiuddin, et al.^42^	2016	13	2.77
16	Hamada, et al.^49^	2019	12	2.56
17	Zeyad, et al.^52^	2019	21	4.48
18	Hamada, et al.^47^	2020	15	3.2
19	Hamada, et al.^48^	2020	12	2.56
20	Al-Mughanam, et al.^53^	2020	42	8.96
21	Salam, et al.^20^	2022	20	4.26
22	Hasan, et al.^19^	2023	18	3.84

**Table 2:** Descriptive statistics of the variables.

Descriptive statistics	Variable						
	C	POFA	CA/FA	W/B	SP	Age	CS
Type of category	Input	Input	Input	Input	Input	Input	Output
Unit	kg/m^3^	kg/m^3^	–	–	kg/m^3^	Days	MPa
Minimum	80.00	0.00	0.81	0.23	0.00	1.00	8.00
Maximum	705.90	336.00	1.53	0.95	21.50	180.00	114.40
Mean	354.49	102.97	1.17	0.41	7.68	35.65	50.32
Standard deviation	111.25	94.84	0.22	0.14	5.12	41.15	23.81
Kurtosis	− 0.46	0.02	− 1.19	0.16	− 0.09	3.74	− 0.52
Skewness	0.10	0.94	− 0.44	0.97	0.38	1.92	0.58

*C* Cement, *POFA* Palm oil fuel ash, *CA* Coarse aggregate, *FA* Fine aggregate, *W* Water, *B* Binder, *SP* Superplasticizer, *CS* Compressive strength.

**Fig. 3:**
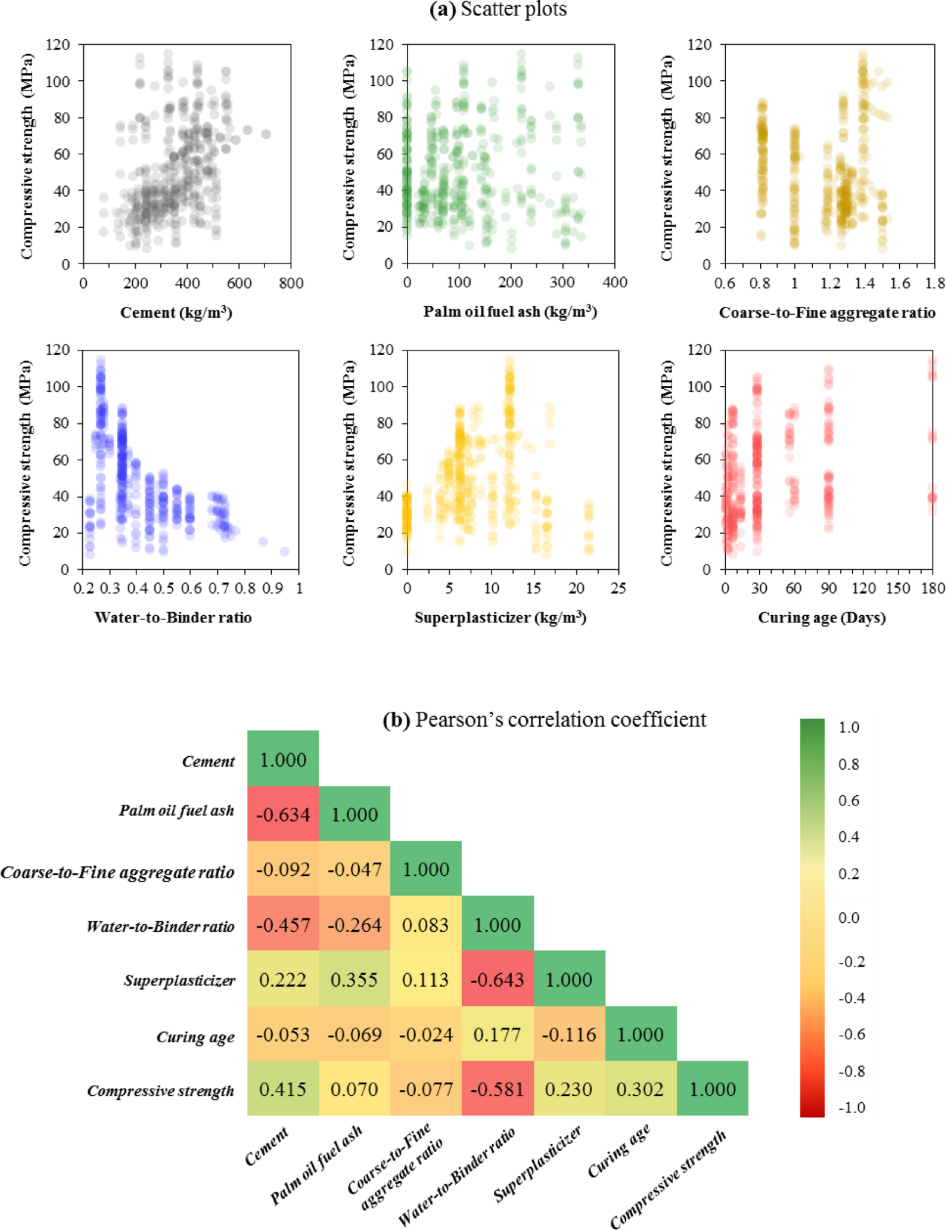
(**a**) Scatter plots of input variables and output variable and (**b**) Pearson’s correlation coefficient.

Prediction accuracy depends on having informative input–output relationships and good coverage of the input distribution to capture a wide range of possibilities. Accordingly, Fig. 3 presents scatter plots of CS versus each input and the Pearson correlation coefficient. Pearson’s correlation quantifies the strength and direction of a linear association between two variables; it is the covariance divided by the product of the standard deviations and ranges from − 1 (perfect negative, indicating inverse correlation) to + 1 (perfect positive, indicating direct correlation)^60^. According to the criterion reported by Shrestha^61^, absolute correlation coefficients below 0.8 (|r|≤ 0.8) generally indicate a low risk of severe multicollinearity. As illustrated in Fig. 3b, all pairwise correlation coefficients among the input features were below this threshold. Therefore, the input dataset did not exhibit evidence of strong multicollinearity, supporting the appropriateness of the selected variables for predictive modeling and subsequent interpretation.

In this dataset, Pearson’s correlation analysis indicates that cement content and W/B are more influential than the other inputs. However, none of the variables shows a strong linear relationship with CS, suggesting that nonlinear AI-based models are more suitable for prediction.

## Methodology

### Artificial neural network (ANN)

An ANN, inspired by the brain’s biological neural system, is a computational method for learning relationships between input and output variables. An ANN typically consists of three main layers, input, hidden, and output. Each layer is composed of interconnected elements known as neurons^62^. Independent variables are introduced to the system through the input layer; the hidden layer (which may include multiple layers) processes these signals via weighted connections and activation functions to capture the underlying relationships; and the output layer produces the dependent variable (compressive strength)^63^. Each connection between neurons carries a numerical weight that indicates the strength of influence from one neuron to the next, while bias terms allow flexible shifting of activation thresholds.

ANNs can model nonlinear relationships between variables that conventional statistical regressions often fail to capture. Their capacity to learn from experimental data enables them to generalize and represent the complex, nonlinear behavior of different mixtures. This flexibility makes ANNs well suited for predicting the mechanical properties of concrete, such as compressive strength.

**Fig. 4:**
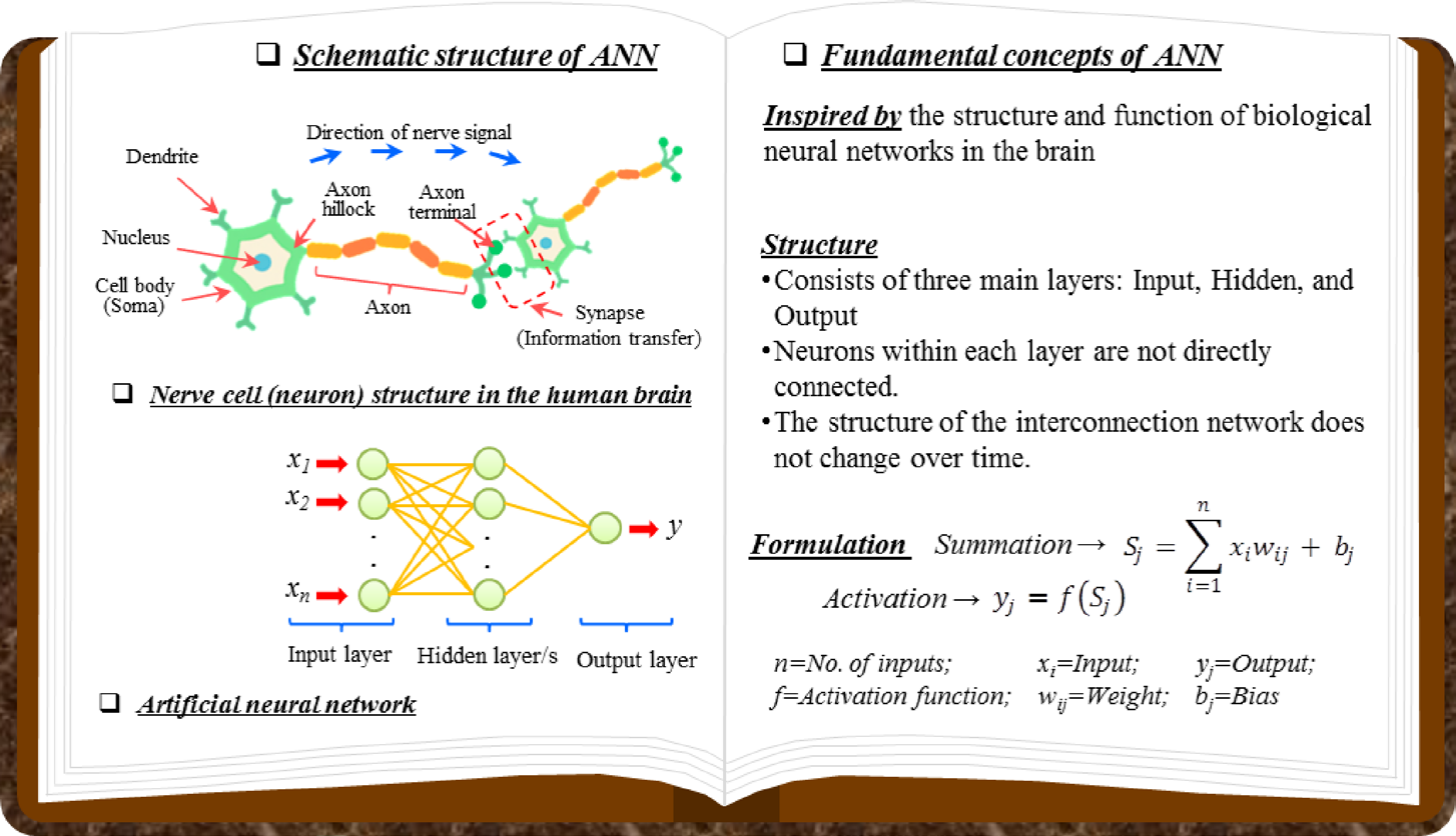
Schematic representation of the ANN architecture.

The overall structure and signal flow of the ANN used in this study are illustrated in Fig. 4, which schematically shows the interconnection of neurons across the input, hidden, and output layers.

### Biogeography-based optimization (BBO)

BBO is a metaheuristic algorithm that operates on populations and is founded on the distribution of biological species across various geographical settings. Simon^64^ proposed the method that employs the mechanisms of migration, mutation, and natural selection observed in ecosystems to identify optimal solutions inside intricate, multidimensional landscapes. In BBO, each potential solution, or habitat, is a possible resolution to the optimization problem. The habitat suitability index (HSI) assesses habitat quality. Habitats exhibiting elevated HSI values are superior, whereas those with diminished HSI values are inferior^65^. Information sharing between solutions occurs through the migration process. Habitats with high HSI tend to share features (called suitability index variables, SIVs) with others through emigration, while those with low HSI improve by immigration, accepting information from better habitats. This mechanism allows promising solutions to influence the rest of the population and guides the search toward the global optimum. To maintain diversity and avoid premature convergence, BBO also employs a mutation operator that randomly alters SIVs in some habitats. Mutation introduces new traits into the population, enhancing global exploration while preserving the balance between exploration and exploitation. Because BBO does not require gradient information, it is particularly efficient for nonlinear, discontinuous, or noisy problems such as optimizing machine-learning models. Its simplicity, few control parameters, and strong global search capability make it a popular optimizer for coupling with predictive algorithms^66^.

In this study BBO was adopted because it has been reported as an effective metaheuristic method for neural network training and other nonlinear optimization problems. BBO uses migration-based information sharing among candidate solutions, which can improve global search performance and help reduce premature convergence to local optima. In contrast to gradient-based procedures, it does not require derivative information, making it suitable for complex search spaces. Previous studies have also shown that BBO can perform competitively compared with other optimizers such as PSO and GA, particularly in multilayer perceptron training^65,66^. Therefore, BBO was considered a suitable optimizer for enhancing ANN-based compressive strength prediction in the present work.

**Fig. 5:**
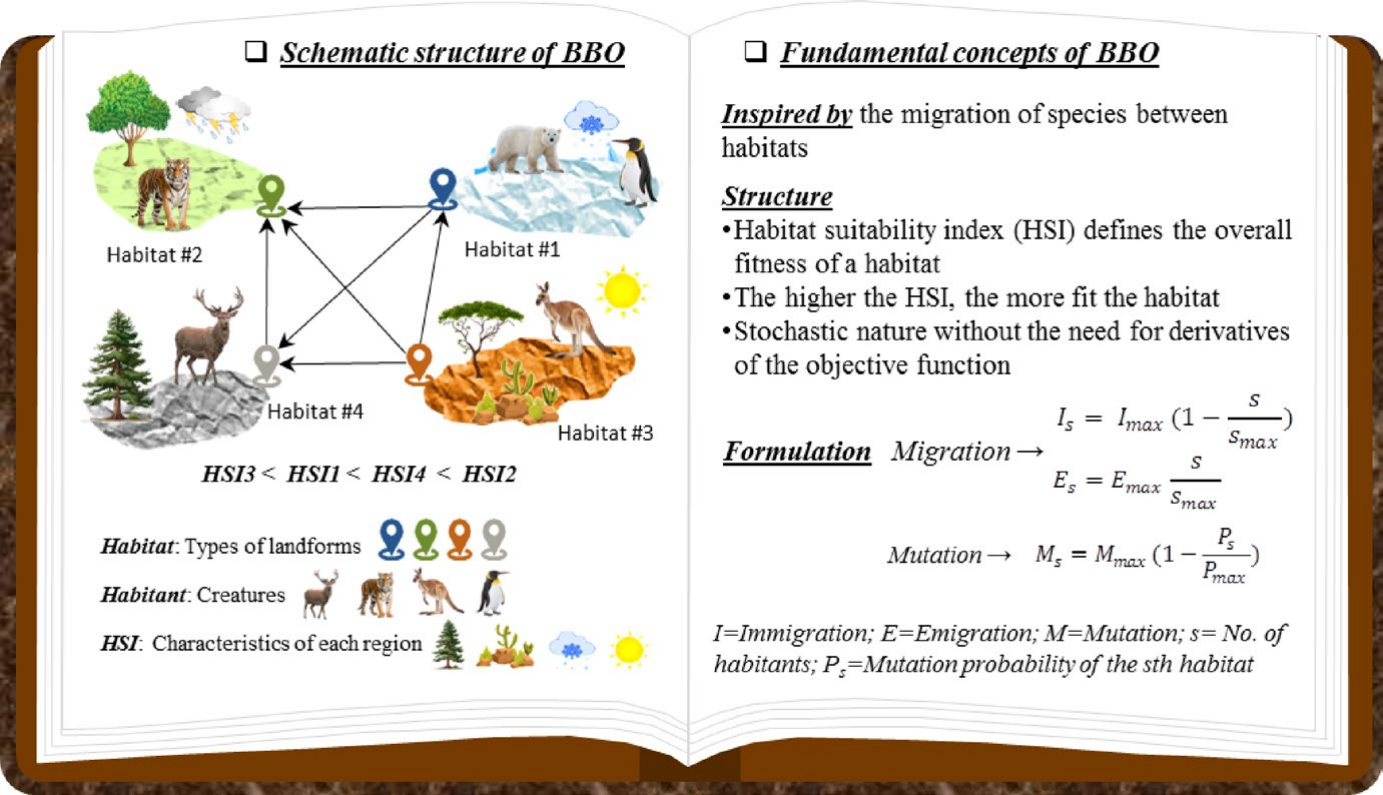
Schematic structure and fundamental concepts of the BBO algorithm.

The conceptual framework and working principles of the BBO algorithm are illustrated in Fig. 5, which shows how species migrate among habitats of different suitability and how migration and mutation collectively drive optimization.

## Model development

In this study, two predictive models were developed to estimate the compressive strength of POFA concrete mixtures: (i) an individual ANN model and (ii) a hybrid ANN-BBO model. Developing both individual and hybrid frameworks allowed for a comparative evaluation of how metaheuristic optimization enhances the predictive capability and stability of standalone models.

Before training, all input and output variables were normalized using the Z-score normalization technique to eliminate the effect of differing data magnitudes and to improve numerical stability during learning^67^. This normalization transforms each variable into a standard scale with zero mean and unit variance according to Eq. (1):

1 $$Z - score = \frac{{{\text{Raw }}\;{\mathrm{data}} - {\text{ Mean}}}}{{{\mathrm{Standard}}\;{\mathrm{deviation}}}}$$

To ensure reliable model evaluation and avoid overfitting, the dataset of 469 samples was divided using the three-way holdout method^68^ into three subsets: 70% (329 samples) for training, 15% (70 samples) for validation, and 15% (70 samples) for testing. The training set was used to update the model parameters, the validation set to monitor convergence and avoid overfitting, and the testing set to evaluate final performance on unseen data. The ANN model was trained using the Levenberg–Marquardt (LM) backpropagation algorithm, which combines the advantages of the Gauss–Newton and gradient descent methods^69,70^. LM is widely regarded as one of the most efficient algorithms for function approximation problems in civil engineering applications because of its rapid convergence and high stability when handling nonlinear datasets^71,72^. The hyperbolic tangent (*tanh*) activation function was adopted for the hidden layer. This function is known for its smooth nonlinearity and strong ability to capture complex input–output relationships. Previous comparative studies have shown that *tanh* activation generally provides higher accuracy and faster convergence than sigmoid or ReLU functions for predicting concrete properties^73,74^.

The initial ANN architecture was defined as 6–H–1, where 6 represents the number of input variables, H denotes the number of hidden neurons, and 1 represents the output neuron for CS. The optimal number of hidden neurons was determined by comparing validation errors across different network architectures. This optimization process, discussed in the following section, ensured that the final model architecture achieved the best balance between accuracy and generalization.

### Model evaluation

The reliability and predictive accuracy of the models were assessed using standard statistical metrics. The formulas and ideal ranges/values for each metric (R^2^, A10-index, MAE, RMSE, RRMSE, VAF, and the objective function) are summarized in Table 3.

**Table 3:** The statistical indicators used to evaluate the proposed models.

Metric brief description formula	Range, ideal value
Coefficient of determination (R^*2*^)	(0, 1) 1
The metric used to evaluate how well the model explains the variability of the response variable	
$$R^{2} = \left( {\frac{{\mathop \sum \nolimits_{{{\mathrm{i}} = 1}}^{T} \left( {CS_{mi} - \overline{{CS_{m} }} } \right)\left( {CS_{pi} - \overline{{CS_{p} }} } \right)}}{{\sqrt {\left[ {\mathop \sum \nolimits_{{{\mathrm{i}} = 1}}^{T} \left( {CS_{mi} - \overline{{CS_{m} }} } \right)^{2} } \right]\left[ {\mathop \sum \nolimits_{{{\mathrm{i}} = 1}}^{T} \left( {CS_{pi} - \overline{{CS_{p} }} } \right)^{2} } \right]} }}} \right)^{2}$$	
a10-index	(0, 100) 100
The proportion of data records whose predicted values deviate by ± 10% from the actual values	
$$a10 = \frac{m10}{T} \times 100$$	
Mean absolute error (MAE)	(0, $+\infty$) 0
The average magnitude of the errors between the predicted and actual values, regardless of their direction	
$$MAE = \frac{1}{T}\mathop \sum \limits_{{{\mathrm{i}} = 1}}^{T} \left| {CS_{mi} - CS_{pi} } \right|$$	
Root mean squared error (RMSE)	(0, $+\infty$) 0
The measure of how widely errors are dispersed around the line of best fit	
$$RMSE = \sqrt {\frac{1}{T}\mathop \sum \limits_{{{\mathrm{i}} = 1}}^{T} \left( {CS_{mi} - CS_{pi} } \right)^{2} }$$	
Relative root mean squared error (RRMSE)	Excellent < 0.05 0.05 < Good < 0.1 0.1 < Fair < 0.15 Poor > 0.15
The RRMSE of the prediction error checks the accuracy of a model compared to the size of the real data. This lets you compare how well models work at different scales	
$$RRMSE = \frac{RMSE}{{\overline{{CS_{m} }} }}$$	
Variance accounted For (VAF)	(0, 100) 100%
The metric to describe the variability of the dataset by the model	
$$VAF = \left( {1 - \frac{{var\left( {CS_{m} - CS_{p} } \right)}}{{var\left( {CS_{m} } \right)}}} \right) \times 100$$	
Objective function (OBJ)	(0, $+\infty$) 0
The OBJ serves as a statistical metric composed of several statistical indicators (RMSE, MAE, and R) for training, validating and testing data	
$$\begin{aligned} OBJ = & \left( {\frac{{T_{Tr} - T_{Va} - T_{Te} }}{T}.\frac{{RMSE_{Tr} + MAE_{Tr} }}{{R_{Tr} + 1}}} \right) \\& \quad + \left( {\frac{{2T_{Va} }}{T}.\frac{{RMSE_{Va} + MAE_{Va} }}{{R_{Va} + 1}}} \right) \\ &\quad + \left( {\frac{{2T_{Te} }}{T}.\frac{{RMSE_{Te} + MAE_{Te} }}{{R_{Te} + 1}}} \right) \\ \end{aligned}$$	

*T*, *T*_*Tr*_, *T*_*Va*_, and *T*_*Te*_ are the number of total, training, validating, and testing data. ${{CS}_{m}}_{i}$ and ${{CS}_{p}}_{i}$ = The measured and predicted CS of the *i*th data, respectively. $\overline{{CS }_{m}}$ and $\overline{{CS }_{p}}$ = The average of total ${{CS}_{m}}_{i}$ and ${{CS}_{p}}_{i}$, respectively. *m10* = The number of data in which their ${{CS}_{m}}_{i}$/${{CS}_{p}}_{i}$ ratio fits in the range of 0.90–1.10.

To obtain a more reliable evaluation of model performance, the dataset containing 469 composite mixtures was analyzed using a tenfold cross-validation procedure during model development. In k-fold cross-validation, the dataset is randomly divided into k approximately equal folds. For each iteration, one fold is retained for validation, whereas the remaining k-1 folds are used for training. This procedure is repeated k times so that each fold is used once for validation, and the average performance across all iterations is then calculated^75^. In the present study, the training–validation dataset was partitioned into ten folds, such that 90% of the data were used for training and 10% for validation in each iteration. The validation errors obtained from the ten runs were averaged to estimate model generalization during development. The final developed models were then assessed on a separate test set to further examine their predictive capability on unseen data. This combined procedure provides a more robust estimate of model performance and reduces the bias that may arise from a single holdout split.

## Results and discussion

**Fig. 6:**
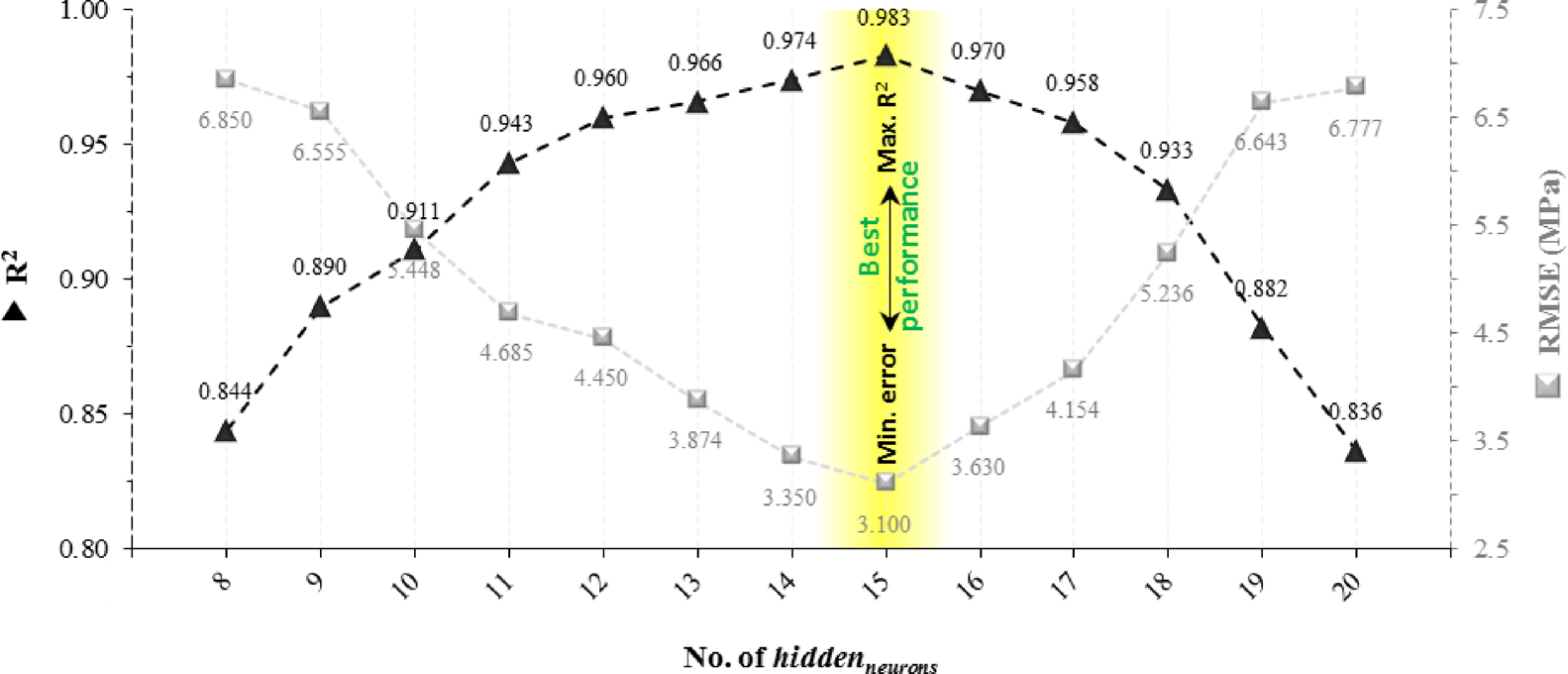
Comparison of different number of hidden layer neurons.

The model was evaluated using hidden-layer sizes ranging from 8 to 20 neurons to identify the optimal architecture for the 6–H–1 network. To guarantee a fair comparison of performance, all models were run on the same dataset. With the highest R^2^ (0.983) and the lowest RMSE (3.1 MPa), the model with 15 hidden neurons produced the best results, as seen in Fig. 6. As a result, the 6–15-1 architecture was chosen as the final configuration to predict POFA concrete’s compressive strength.

**Fig. 7:**
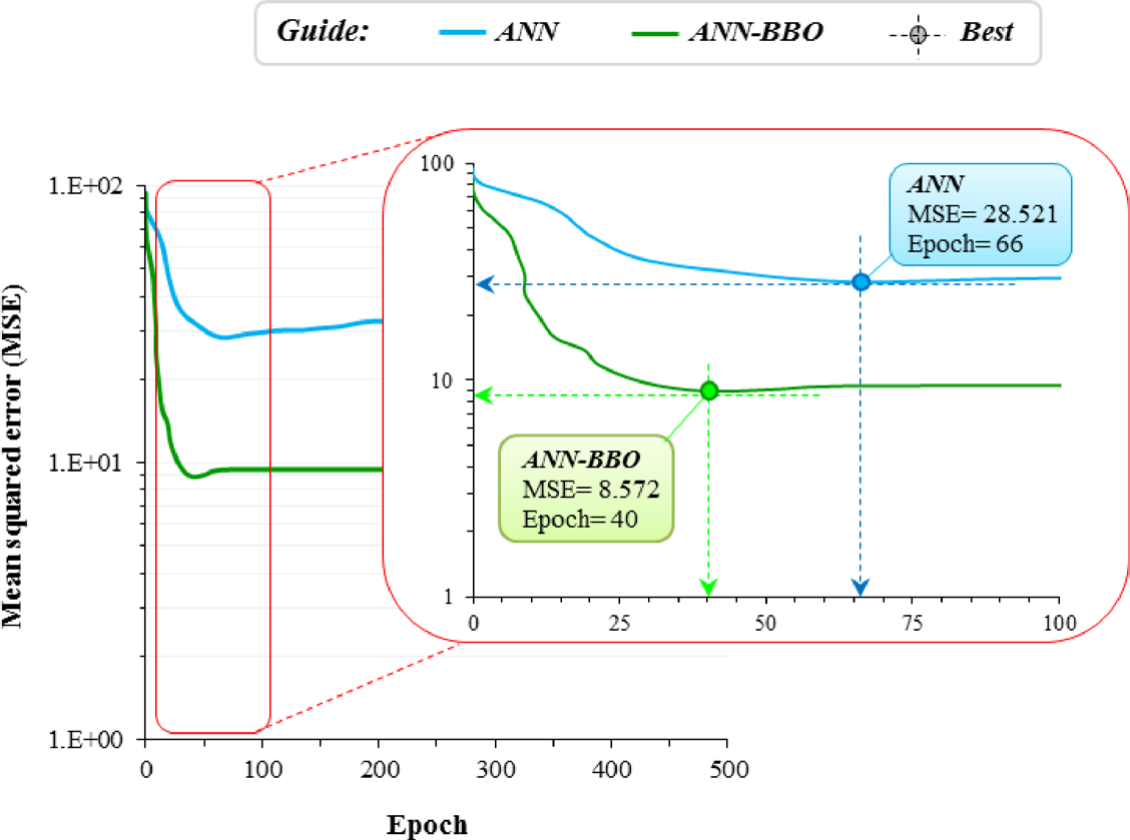
Convergence curves of the performance of models.

Figure 7 compares the convergence behavior of the ANN and ANN-BBO models in terms of mean MSE over the training epochs. As shown, ANN reached its best performance at epoch 66 with an MSE of 28.521, whereas the ANN-BBO model attained a substantially lower MSE of 8.572 at epoch 40. These results indicate that BBO-based optimization enhanced the training efficiency of the neural network by reaching a lower error in fewer epochs. This improved convergence behavior suggests that ANN-BBO provided a more effective search for favorable network parameters than the conventional ANN training procedure.

Finally, Table 4 summarizes the settings of selected parameters for ANN and BBO models.

**Table 4:** Parameter settings for models.

Model	Parameter		Setting
ANN	Data dividing (%-No. of data)	Training	70%–329
		Validating	15%–70
		Testing	15%–70
	No. of hidden layer/nodes		1/15
	Learning algorithm		Levenberg–Marquardt
	Transfer function		Tanh
	No. of iterations		500
BBO	Population size		100
	Max immigration and emigration rate		1
	Habitat modification		1
	Mutation probability		0.005
	No. of generations (iterations)		500

**Fig. 8:**
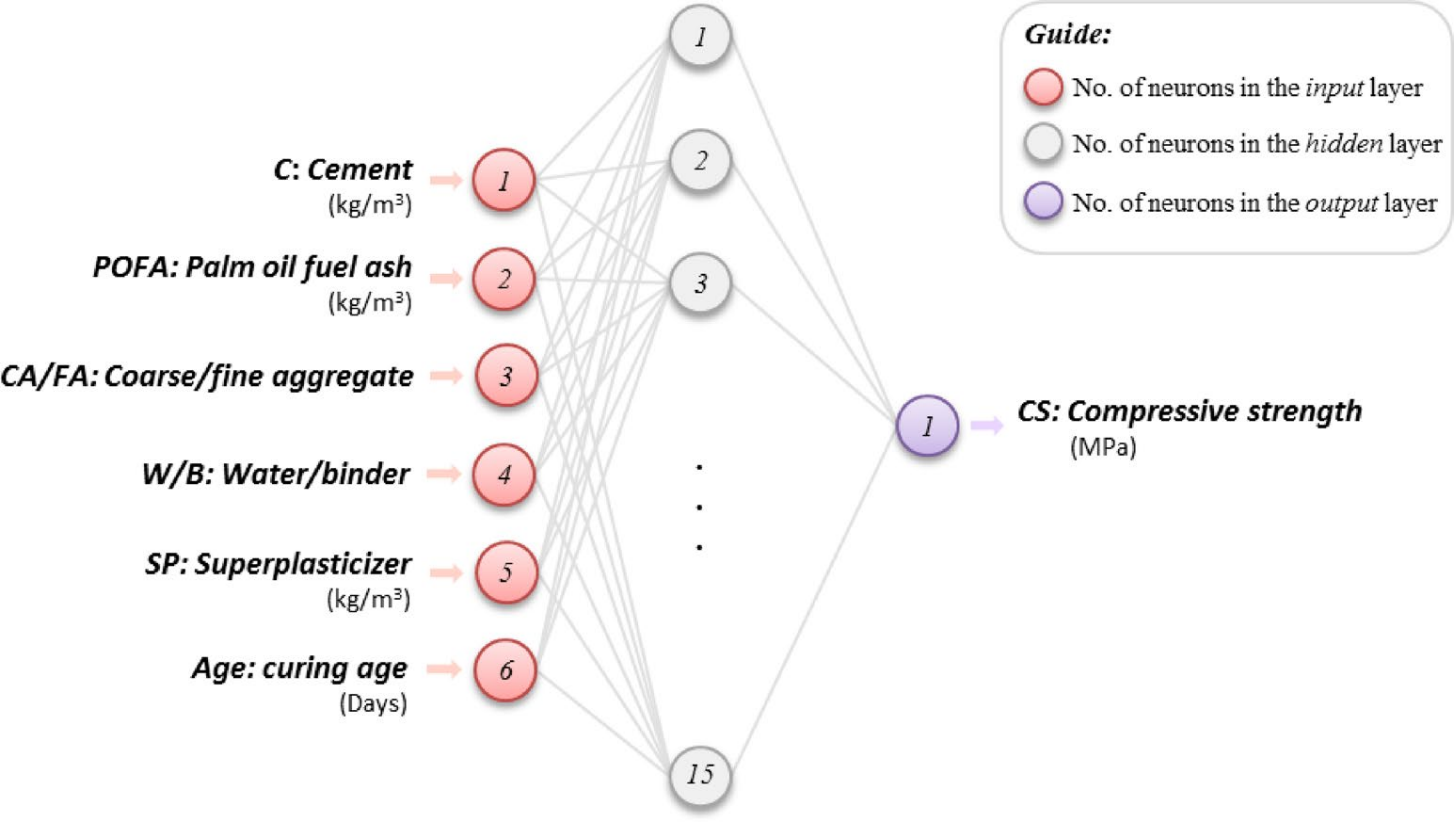
Final structure for model with the best performance.

Figure 8 shows the corresponding schematic of the chosen model structure, with six neurons representing the mix parameters in the input layer, fifteen neurons in the hidden layer, and one neuron representing the predicted compressive strength in the output layer.

### Regression analysis

**Fig. 9:**
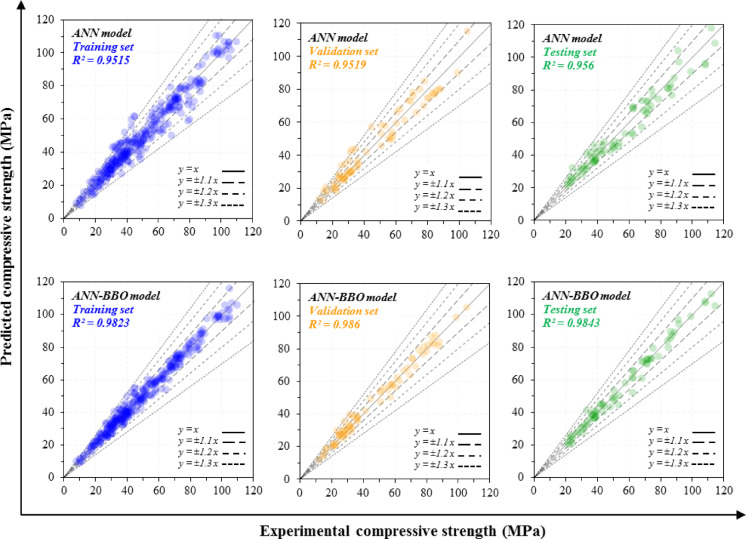
Experimental and predicted compressive strength values for training, validation, and testing datasets using ANN and ANN-BBO models.

The performance of the predictive models, ANN and ANN-BBO, was evaluated using the training, validation, and testing datasets. As Fig. 9 shows, the correlation between experimental and predicted compressive strength is strong: the points cluster near the y = x line with narrow deviation bands, indicating that both models capture the nonlinear relationship between input variables and compressive strength. However, the ANN-BBO shows better performance than the individual ANN, with R^2^ values of 0.9823, 0.986, and 0.9843 for the training, validation, and testing sets, respectively, compared with 0.9515, 0.9519, and 0.956 for the ANN. This demonstrates a clear improvement in predictive performance for ANN-BBO over ANN.

### Error histogram plot

**Fig. 10:**
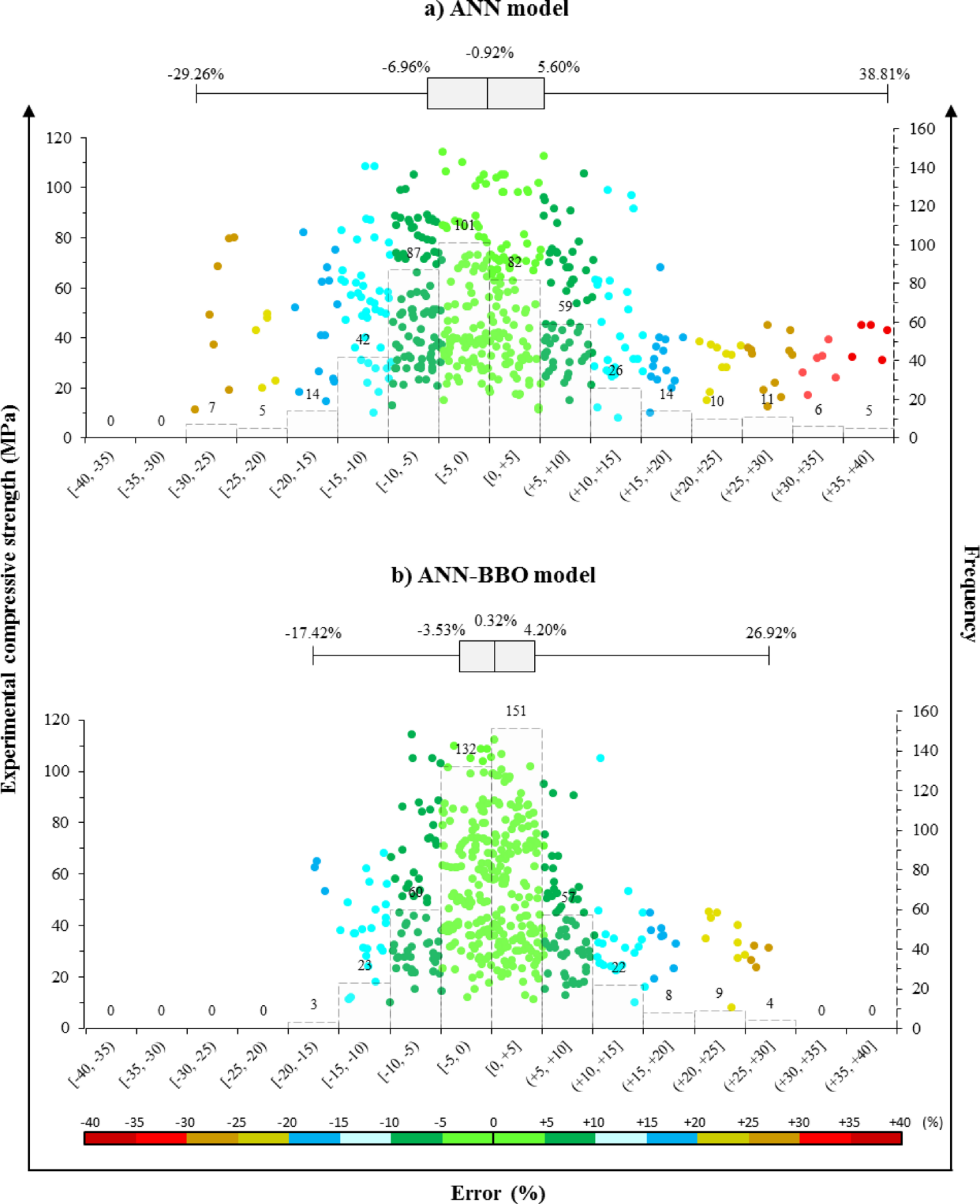
Error distribution of the ANN and ANN-BBO models.

The distribution of prediction errors for the ANN and ANN-BBO models for the compressive strength of POFA is shown in Fig. 10. Each histogram and scatter plot illustrates the deviation error between the experimental and predicted compressive strength values for the training, validation, and testing sets. In both models, most data points are clustered around the zero-error line, indicating that the predictions are generally unbiased and reasonably accurate.

However, the ANN-BBO model exhibits a narrower and more symmetric error distribution: about 39% of the errors for the ANN model fall within the range [− 5%, 5%], whereas more than 60% of the results for ANN-BBO lie in the same interval, confirming the higher predictive accuracy of the hybrid model. This conclusion is also supported by the boxplots of percentage error. For the ANN-BBO model, 50% of the data lie between approximately -3.53% and + 4.20%, with a median error of 0.32%, while the same 50% of the errors for the ANN model lie in a wider range, from about − 6.96% to + 5.60%, with a median error of − 0.92%. These results demonstrate that the hybrid ANN-BBO model achieves greater precision and stability, effectively reducing random deviations and improving overall predictive accuracy compared with the ANN.

**Fig. 11:**
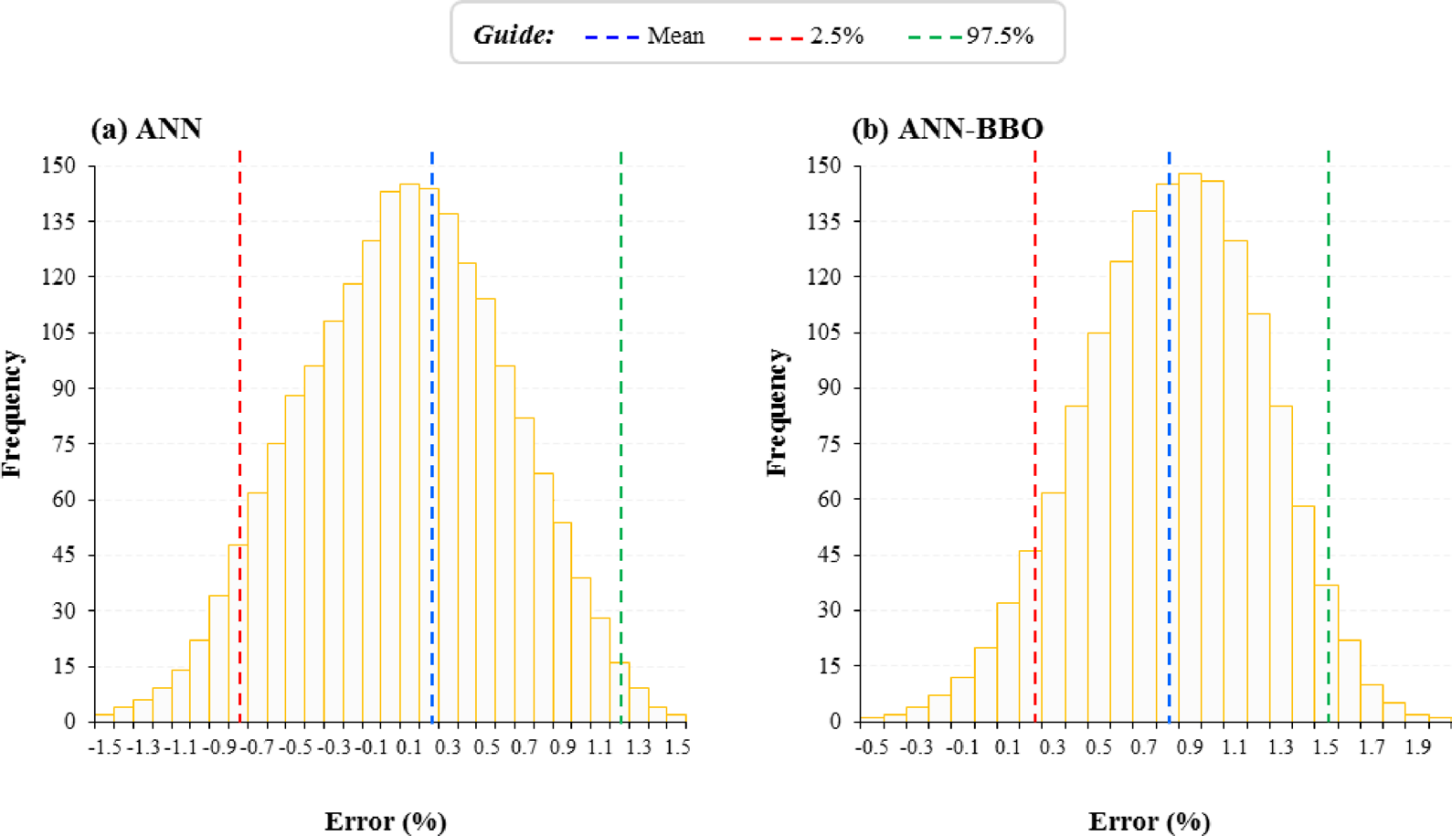
Bootstrap error distributions for ANN and ANN-BBO models based on 1000 resamples.

Figure 11 illustrates the empirical distribution of prediction errors obtained from 1000 bootstrap resamples for both ANN and ANN-BBO models. The distributions for both models are bell-shaped and almost symmetric, which indicates stable sampling behavior. Table 5 also presents the corresponding results for the bootstrap distribution. As shown in Fig. 11 and Table 5, for the ANN model the mean percentage error is 0.22%, with a 95% confidence interval (CI) ranging from − 0.82 to 1.26%, giving a CI width of 2.08%. On the other hand, the ANN-BBO model yields a mean error of 0.85%, with a narrower 95% confidence interval ranging from 0.23 to 1.51%, and a CI width of 1.28%. The bootstrap analysis showed that the ANN-BBO model had a slightly higher mean prediction error than the standalone ANN model; however, its confidence interval was noticeably narrower. This indicates that, although the average bootstrap error of ANN-BBO was marginally larger, its predictive performance was more consistent across bootstrap resamples and less sensitive to sampling variability. In contrast, the ANN model yielded a lower mean error but exhibited a wider CI, suggesting greater variation in performance across resampled datasets. This pattern is consistent with the bias–variance trade-off commonly observed in machine learning, where a model with slightly higher average error may nonetheless provide more stable predictions. Therefore, the bootstrap results should not be interpreted as showing uniformly better accuracy for ANN-BBO; rather, they indicate that ANN-BBO offered improved performance and stability, whereas ANN showed slightly lower average bootstrap error.

**Table 5:** Summary of bootstrap statistics for ANN and ANN-BBO models, including mean error.

Metric	ANN	ANN-BBO
Mean (%)	0.22	0.85
2.5th percentile	− 0.82	0.23
97.5th percentile	1.26	1.51
CI width (%)	2.08	1.28

The narrower confidence interval observed for the ANN-BBO model indicates reduced sampling variability and tighter clustering of bootstrap estimates. This suggests that ANN-BBO provides more reliable and consistent predictions under repeated resampling.

### Evaluating performance metrics

**Fig. 12:**
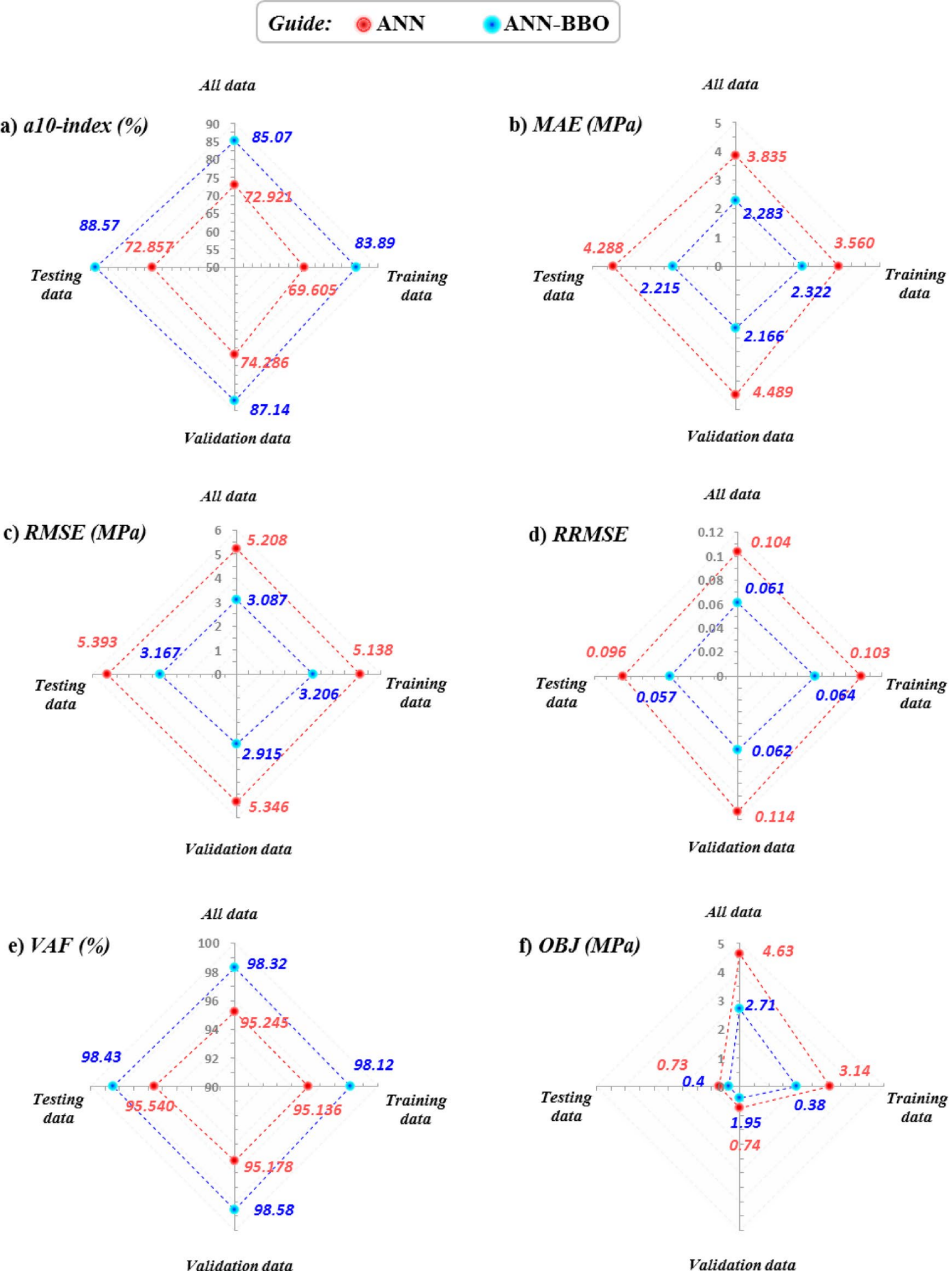
Comparison of ANN and ANN-BBO performance across datasets.

To compare the two models more clearly, Fig. 12 presents a sensitivity analysis summary of the statistical metrics, and Table 6 lists the corresponding values. Figure 12a–f show, respectively, the a10-index, MAE, RMSE, RRMSE, VAF, and OBJ. Results are reported for the all, training, validation, and testing subsets for both ANN and ANN-BBO. Note that the ideal behavior is a10-index and VAF close to 100%, and MAE, RMSE, RRMSE, and OBJ close to 0.

**Table 6:** Numerical values of evaluation metrics for ANN and ANN-BBO on all, training, validation, and testing sets.

Model	Data set	Metrics					
		a10-index (%)	MAE (MPa)	RMSE (MPa)	RRMSE	VAF (%)	OBJ (MPa)
ANN	Training	69.60	3.560	5.138	0.103	95.14	3.14
	Validation	74.29	4.489	5.346	0.114	95.18	0.74
	Testing	72.86	4.288	5.393	0.096	95.54	0.73
	All	72.92	3.835	5.208	0.104	95.24	4.63
ANN-BBO	Training	83.89	2.322	3.206	0.064	98.12	1.95
	Validation	87.14	2.166	2.915	0.062	98.58	0.38
	Testing	88.57	2.215	3.167	0.057	98.43	0.40
	All	85.07	2.283	3.087	0.061	98.32	2.71

As can be seen from Fig. 12 and Table 6, the ANN-BBO model outperformed the ANN model, showing lower errors in all metrics. For example, the RMSE (Fig. 12c) values obtained from ANN-BBO for all, training, testing, and validation data are 3.087, 3.206, 3.167, and 2.915 MPa, respectively, while the corresponding RMSE values for ANN are 5.208, 5.138, 5.393, and 5.346 MPa, respectively. Additionally, the a10-index shows higher values for ANN-BBO than for the ANN model, reflecting the percentage of data records that satisfy 0.9 < experimental CS/predicted CS < 1.1.

**Fig. 13:**
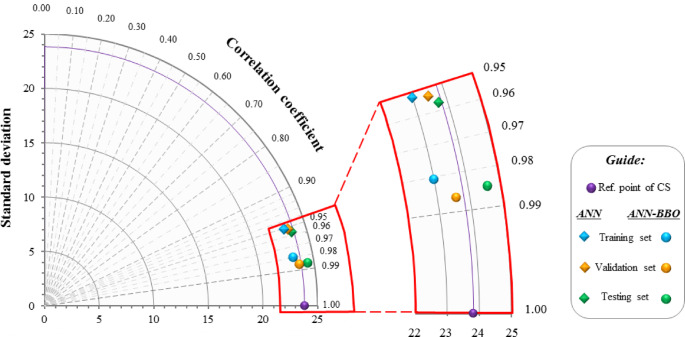
Taylor diagram for the ANN and ANN-BBO models.

Figure 13 presents a Taylor diagram comparing the performance of the ANN and ANN-BBO models. The diagram simultaneously displays the correlation coefficient and standard deviation, providing a compact visual summary of model accuracy across the training, validation, and testing datasets. Although both models show reliable performance in predicting the compressive strength of POFA, the ANN-BBO points lie closer to the reference, indicating higher correlation and lower deviation from the experimental data. The ANN points lie farther from the reference, reflecting slightly greater variability and error. This graphical illustration confirms the consistency, accuracy, and stability of the hybrid ANN-BBO model compared with the ANN, in agreement with the earlier performance results.

**Fig. 14:**
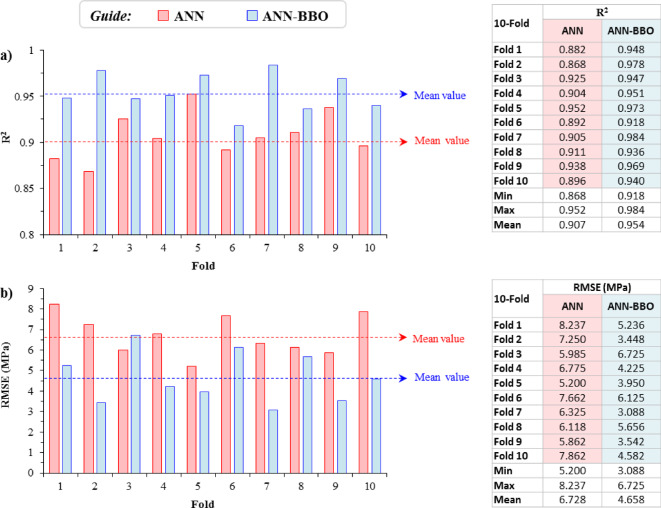
Cross-validation results for ANN and ANN-BBO models.

To evaluate model robustness and reduce the bias associated with a single data split, a ten-fold cross-validation procedure was conducted. Figure 14 summarizes the R^2^ and RMSE values obtained for the ANN and ANN-BBO models over the ten folds, with the dotted lines representing the corresponding mean values. The ANN model exhibited R^2^ values ranging from 0.868 to 0.952, with a mean of 0.907, while the ANN-BBO model achieved R^2^ values between 0.918 and 0.984, with a higher mean of 0.954. In terms of error, the RMSE values for ANN varied from 5.200 to 8.237 MPa, with an average of 6.728 MPa. For ANN-BBO, the RMSE ranged from 3.088 to 6.725 MPa, with a lower average of 4.658 MPa. The average RMSE of ANN-BBO was therefore about 30.8% lower than that of ANN (see Fig. 14). These results show that the ANN-BBO model maintained better predictive accuracy across the cross-validation folds and provided a more reliable estimate of generalization performance.

### Contribution of variables

**Fig. 15:**
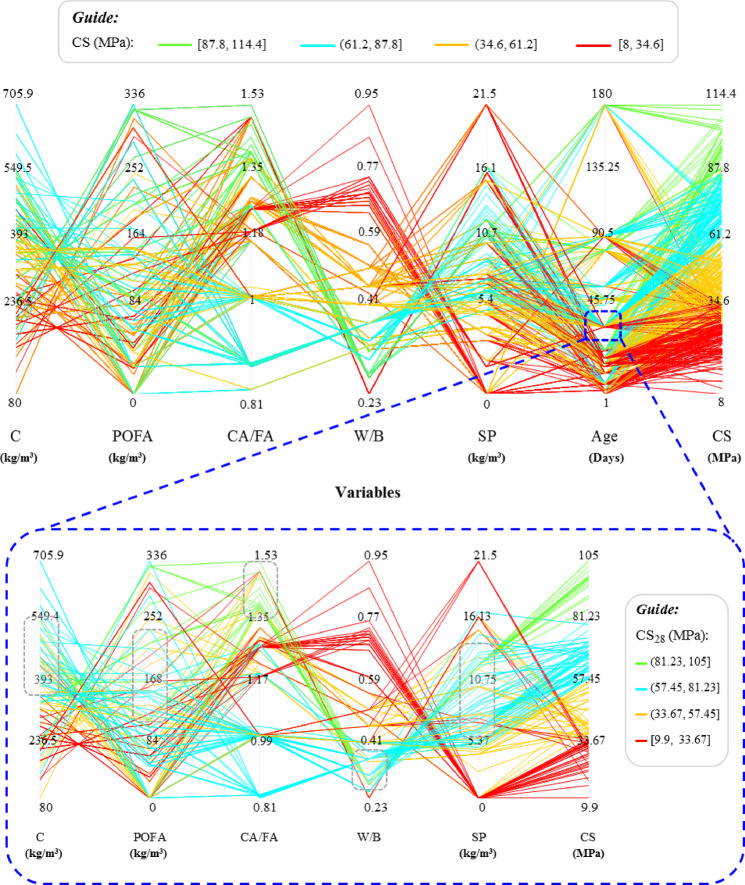
Parallel coordinate plot for the ANN-BBO model showing the relationships between input variables and predicted compressive strength.

The parallel coordinate plot in Fig. 15 is mainly used as an exploratory check of how the ANN-BBO model behaves across the full input space. Each polyline represents one mixture, and the colors indicate different compressive strength ranges. As shown in Fig. 15, high-strength mixtures (green and cyan lines) tend to cluster around higher cement content, lower water-to-binder ratios, and moderate POFA and SP dosages. Low-strength mixtures (yellow and red lines) are more frequently associated with higher W/B, lower cement content, and less favorable combinations of aggregates and POFA. The wider spread of the yellow/red trajectories indicates larger variability in poorly proportioned mixtures.

Since the 28-day compressive strength is a key criterion in concrete mix design, the magnified section of Fig. 15 focuses on how each input parameter contributes to CS_28_. The dotted grey rectangles indicate the approximate optimum ranges of the input variables associated with higher 28-day strength. As illustrated in Fig. 15, the optimum ranges for cement, POFA, and superplasticizer contents are roughly 330–550, 110–220, and 6–13 kg/m^3^, respectively, while the favorable ranges for the CA/FA and W/B ratios are about 1.35–1.50 and 0.28–0.35, respectively.

Overall, this visualization confirms that the hybrid ANN-BBO model recognizes meaningful multidimensional patterns in the dataset, effectively separates well-designed mixtures from suboptimal ones, and demonstrates clear sensitivity to the parameters most influential on compressive strength.

**Fig. 16:**
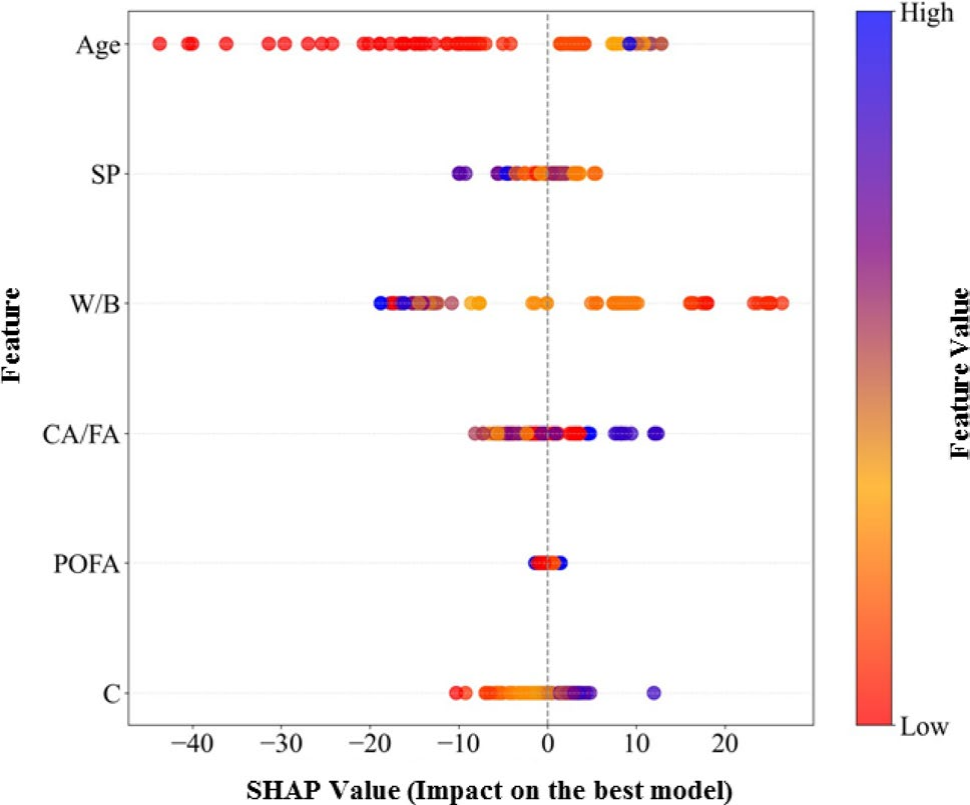
Summary plot of SHAP values.

Figure 16 presents the SHapley Additive exPlanations (SHAP) summary for the ANN-BBO model, showing the relative importance of the input variables and their effects on the predicted 28-day compressive strength. Each point represents an individual mixture, where the horizontal position indicates the SHAP value (i.e., the contribution of a given feature to increasing or decreasing the predicted strength), and the color reflects the magnitude of the feature value.

As shown in Fig. 16, age has the strongest overall influence on compressive strength, with higher curing ages generally contributing positively and lower ages contributing negatively. This result is physically expected because the SHAP analysis was performed on the full dataset, which includes compressive strength measurements at multiple curing ages rather than only 28-day strength. In this context, the strong contribution of age reflects the well-established time-dependent nature of strength development in cementitious materials, governed by hydration progress and microstructural evolution. At the same time, because the dataset spans multiple curing ages, age captures a substantial portion of the overall variation in compressive strength and should therefore be interpreted within the framework of multi-age prediction. W/B is also highly influential, with higher W/B values mostly contributing negatively and lower W/B values contributing positively, consistent with the expected reduction in strength at higher water contents. Cement content (C) and CA/FA exhibit moderate effects; higher cement content generally tends to increase strength, while CA/FA shows a mixed pattern, suggesting nonlinear interactions with other mixture variables. Superplasticizer dosage has a smaller but still observable influence, mainly positive at moderate levels. POFA shows the lowest SHAP magnitude, indicating that its effect is less direct and likely depends strongly on its interactions with other parameters. Overall, the SHAP results suggest that the ANN-BBO model captures relationships that are broadly consistent with established concrete behavior.

### Validating the proposed model by comparison with literature models

To compare the proposed ANN-BBO model with other predictive models, its performance was evaluated against the results reported by Ali et al.^43^, who developed seven different AI models to predict the compressive strength of concrete containing POFA. These methods included LSSVM, LGBM, XGB, hybrid XGB-LGBM, ANN, and GEP. They trained and tested these models on a POFA database, and their performance metrics are summarized in Table 7 alongside the results of the ANN-BBO model proposed in the current study.

**Table 7:** Comparison of the proposed ANN-BBO model with other prediction models for predicting CS of POFA concrete.

Authors (year)	No. data used	The developed model	Statistical metrics		
			R^2^ achieved	MAE achieved (MPa)	RRMSE achieved
Current study	469	ANN-BBO	0.983	2.28	0.061
Ali et al.^43^	407	LSSVM	0.845	9.05	0.217
		Bagging	0.952	4.85	0.121
		LGBM	0.955	4.22	0.117
		XGB	0.962	3.88	0.112
		Hybrid XGB-LGBM	0.976	3.11	0.080
		ANN	0.968	3.82	0.099
		GEP	0.499	11.1	0.464

As shown in Table 7, among the models proposed by Ali et al.^43^, the hybrid XGB-LGBM and ANN models performed better than the others, with R^2^ values of 0.976 and 0.968, respectively, and relatively low MAE values of 3.11 MPa and 3.82 MPa compared to other models. However, the ANN-BBO model still shows better performance: it achieves a higher R^2^ of 0.983, and both MAE and RRMSE are lower than those reported for the hybrid XGB-LGBM and ANN models. It is also important to note that these improvements were achieved on a larger database. While Ali et al.^43^ used 407 data points, the present study employed 469 mixtures (i.e., more than a 15% increase in dataset size). These results suggest that the developed model provides improved predictive performance relative to previously reported methods, while being trained on a larger dataset.

*LSSVM* Least square support vector machine, *LGBM* Light gradient boosting machine, *XGB* Extreme gradient boosting, *GEP* Gene expression programming.

## Limitations and implications

A key limitation of the present database is that it was compiled from 22 independent studies with unavoidable differences in specimen dimensions, curing conditions, testing procedures, and POFA pre-treatment methods. Although these studies were combined to enable broader data-driven modeling, such study-level heterogeneity was not fully controllable because several metadata fields were unavailable or inconsistently reported in the source literature. As a result, part of the variation captured by the models may reflect inter-study differences rather than mixture design variables alone. Therefore, the predictive trends reported herein should be interpreted within the context of the compiled literature dataset rather than as fully harmonized universal relationships. Future work should prioritize database expansion with standardized metadata reporting and apply grouped or study-aware sensitivity analyses to better isolate the effect of inter-study variability on model performance. Although the compiled database was derived from multiple independent studies and therefore contains inherent inter-study variability, several measures were taken to reduce potential bias. The collected data were carefully screened and standardized in terms of parameter definitions and measurement units before model development. In addition, the database covers a broad range of input variables and compressive strength values, which helps reduce overrepresentation of any single mixture category or experimental condition. Furthermore, bootstrap resampling showed relatively stable prediction errors and narrow confidence intervals, suggesting that model performance was not dominated by a particular subset of the heterogeneous dataset.

From a theoretical perspective, this study suggests that a hybrid metaheuristic model can improve compressive strength prediction relative to a standalone ANN model for concrete datasets. It also demonstrates the benefit of integrating predictive modeling with interpretability analysis to clarify the roles of mixture and curing variables. From a practical perspective, the proposed model may assist researchers and engineers in the rapid estimation and preliminary assessment of POFA-based concrete mixtures, although its use should remain within the scope of the compiled dataset and be supported by experimental validation.

## Conclusions and future directions

This study developed data-driven models to predict the compressive strength of concrete incorporating POFA using a comprehensive database of 469 mixtures. Two predictive approaches, ANN and ANN-BBO, were constructed and evaluated using a wide range of statistical indicators, error analyses, and reliability assessments. Based on the results and analyses carried out in this study, the main conclusions can be summarized as follows:

- Regression analysis indicated that ANN-BBO achieved better predictive performance than the standalone ANN, with R^2^ values of 0.9823, 0.986, and 0.9843 for the training, validation, and testing sets, respectively, compared with 0.9515, 0.9519, and 0.956 for the ANN.
- The error histogram and bootstrap error distribution showed that ANN-BBO provides more stable predictions, reduced variability, and narrower confidence intervals across all data subsets. Approximately 60% of ANN-BBO predictions fell within the ± 5% error band, compared to roughly 39% for the ANN model, demonstrating markedly improved precision and reduced random deviation. Moreover, the narrower confidence interval width for ANN-BBO (1.28%) compared with ANN (2.08%) confirms its higher accuracy.
- Taylor diagram and sensitivity analyses of statistical metrics including the a10-index, MAE, RMSE, RRMSE, VAF, and OBJ also confirmed that the ANN optimized with BBO performed better than ANN in predicting compressive strength.
- The parallel coordinate analysis showed that the ANN-BBO model can clearly distinguish well-proportioned, high-strength mixtures, characterized by higher cement content, lower W/B ratios, and moderate POFA and SP levels, from low-strength mixtures with less favorable parameter combinations. This visualization confirms that the hybrid model captures meaningful multidimensional patterns and is highly sensitive to the key variables governing compressive strength.
- Validation against seven literature models demonstrated that ANN-BBO showed more consistent results compared to advanced techniques such as LSSVM, XGB, LGBM, hybrid XGB–LGBM, and ANN. Achieving higher R^2^ (0.983) and lower MAE and RRMSE (0.05 and 0.061, respectively), the proposed model showed higher accuracy despite being trained on a larger and more diverse dataset.

While the findings demonstrate the reliability of the hybrid model, several opportunities remain for advancing POFA-based strength prediction. Future research could expand the dataset to better capture mixtures with higher POFA replacement levels. Incorporating additional input features, such as fineness, LOI, and chemical composition may improve model interpretability and robustness. Exploring alternative hybrid or ensemble machine-learning approaches could provide deeper insight into the nonlinear interactions governing POFA-modified concretes. Finally, extending the modeling framework to other performance indicators, such as durability, permeability, shrinkage, and long-term strength, would support broader application of AI tools in designing sustainable concrete.

## Supplementary Information

Below is the link to the electronic supplementary material.

Supplementary Material 1

## Data Availability

The database used in this study is available in the supplementary file.

## Abbreviations

AI: Artificial intelligence
ANN: Artificial neural network
B: Binder
BBO: Biogeography-based optimization
C: Cement
CA: Coarse aggregate
Ca(OH)_2_: Calcium hydroxide
CI: Confidence interval
CS: Compressive strength
C-S-H: Calcium silicate hydrate
FA: Fine aggregate
GB: Gradient boosting algorithms
GEP: Gene expression programming
HSI: Habitat suitability index
LGBM: Light gradient boosting machine
LM: Levenberg-Marquardt
MAE: Mean absolute error
OBJ: Objective function
POFA: Palm oil fuel ash
R^2^: Coefficient of determination
RMSE: Root mean squared error
RRMSE: Relative root mean squared error
SCC: Self-compacting concrete
SCM: Supplementary cementitious material
SIV: Suitability index variable
SP: Superplasticizer
SVM: Support vector machines
VAF: Variance accounted for
W: Water
XGB: Extreme gradient boosting

## Declarations

### Declaration on use of AI-generated text

The authors confirm that artificial intelligence (AI) tools do not generate the content submitted in this work. During its preparation, these tools were only used to enhance language clarity and readability. After using these tools, the authors reviewed and edited the content as needed and took full responsibility for the content of the publication.
